# Characteristics and outcomes of bacteremia among ICU-admitted patients with severe sepsis

**DOI:** 10.1038/s41598-020-59830-6

**Published:** 2020-02-19

**Authors:** Akira Komori, Toshikazu Abe, Shigeki Kushimoto, Hiroshi Ogura, Atsushi Shiraishi, Daizoh Saitoh, Seitaro Fujishima, Toshihiko Mayumi, Toshio Naito, Toru Hifumi, Yasukazu Shiino, Taka-aki Nakada, Takehiko Tarui, Yasuhiro Otomo, Kohji Okamoto, Yutaka Umemura, Joji Kotani, Yuichiro Sakamoto, Junichi Sasaki, Shin-ichiro Shiraishi, Kiyotsugu Takuma, Ryosuke Tsuruta, Akiyoshi Hagiwara, Kazuma Yamakawa, Tomohiko Masuno, Naoshi Takeyama, Norio Yamashita, Hiroto Ikeda, Masashi Ueyama, Satoshi Fujimi, Satoshi Gando, JAAM FORECAST group, Osamu Tasaki, Yasumitsu Mizobata, Hiraku Funakoshi, Toshiro Okuyama, Iwao Yamashita, Toshio Kanai, Yasuo Yamada, Mayuki Aibiki, Keiji Sato, Susumu Yamashita, Kenichi Yoshida, Shunji Kasaoka, Akihide Kon, Hiroshi Rinka, Hiroshi Kato, Hiroshi Okudera, Eichi Narimatsu, Toshifumi Fujiwara, Manabu Sugita, Yasuo Shichinohe, Hajime Nakae, Ryouji Iiduka, Mitsunobu Nakamura, Yuji Murata, Yoshitake Sato, Hiroyasu Ishikura, Yasuhiro Myojo, Yasuyuki Tsujita, Kosaku Kinoshita, Hiroyuki Yamaguchi, Toshihiro Sakurai, Satoru Miyatake, Takao Saotome, Susumu Yasuda, Yasuaki Mizushima

**Affiliations:** 1 Department of General Medicine, Juntendo University, Tokyo, Japan; 2 Health Services Research and Development Center, University of Tsukuba, Tsukuba, Japan; 3 Department of Health Services Research, Faculty of Medicine, University of Tsukuba, Tsukuba, Japan; 4 Division of Emergency and Critical Care Medicine, Tohoku University Graduate School of Medicine, Sendai, Japan; 5 Department of Traumatology and Acute Critical Medicine, Osaka University Graduate School of Medicine, Osaka, Japan; 6 Emergency and Trauma Center, Kameda Medical Center, Kamogawa, Japan; 7 Division of Traumatology, Research Institute, National Defense Medical College, Tokorozawa, Japan; 8 Center for General Medicine Education, Keio University School of Medicine, Tokyo, Japan; 9 Department of Emergency Medicine, School of Medicine, University of Occupational and Environmental Health, Kitakyushu, Japan; 10 Department of Emergency and Critical Care Medicine, St. Luke’s International Hospital, Tokyo, Japan; 11 Department of Acute Medicine, Kawasaki Medical School, Kurashiki, Japan; 12 Department of Emergency and Critical Care Medicine, Chiba University Graduate School of Medicine, Chiba, Japan; 13 Department of Trauma and Critical Care Medicine, Kyorin University School of Medicine, Mitaka, Japan; 14 Trauma and Acute Critical Care Center, Medical Hospital, Tokyo Medical and Dental University, Tokyo, Japan; 15 Department of Surgery, Center for Gastroenterology and Liver Disease, Kitakyushu City Yahata Hospital, Kitakyushu, Japan; 16 Department of Disaster and Emergency Medicine, Kobe University Graduate School of Medicine, Kobe, Japan; 17 Emergency and Critical Care Medicine, Saga University Hospital, Saga, Japan; 18 Department of Emergency and Critical Care Medicine, Keio University School of Medicine, Tokyo, Japan; 19 Department of Emergency and Critical Care Medicine, Aizu Chuo Hospital, Aizuwakamatsu, Japan; 20 Emergency & Critical Care Center, Kawasaki Municipal Kawasaki Hospital, Kawasaki, Japan; 21 Advanced Medical Emergency & Critical Care Center, Yamaguchi University Hospital, Ube, Japan; 22 Department of Emergency Medicine, Niizashiki Chuo General Hospital, Niiza, Japan; 23 Division of Trauma and Surgical Critical Care, Osaka General Medical Center, Osaka, Japan; 24 Department of Emergency and Critical Care Medicine, Nippon Medical School, Tokyo, Japan; 25 Advanced Critical Care Center, Aichi Medical University Hospital, Nagakute, Japan; 26 Advanced Emergency Medical Service Center, Kurume University Hospital, Kurume, Japan; 27 Department of Emergency Medicine, Teikyo University School of Medicine, Tokyo, Japan; 28 Department of Trauma, Critical Care Medicine, and Burn Center, Japan Community Healthcare Organization, Chukyo Hospital, Nagoya, Japan; 29 Division of Acute and Critical Care Medicine, Hokkaido University Graduate School of Medicine, Sapporo, Japan; 30 Department of Acute and Critical Care Medicine, Sapporo Higashi Tokushukai Hospital, Sapporo, Japan; 31 Japan Association for Acute Medicine, Tokyo, Japan; 32 Nagasaki University Hospital, Nagasaki, Japan; 33 Osaka City University Hospital, Osaka, Japan; 34 Tokyobay Urayasu Ichikawa Medical Center, Chiba, Japan; 35 Aso Iizuka Hospital, Fukuoka, Japan; 36 Tomei Atsugi Hospital, Atsugi, Japan; 37 Hiratsuka City Hospital, Hiratsuka, Japan; 38 National Hospital Organization Sendai Medical Center, Sendai, Japan; 39 Ehime University Hospital, Ehime, Japan; 40 Okayama University Hospital, Okayama, Japan; 41 Tokuyama Central Hospital, Shunan, Japan; 42 Fukuyama City Hospital, Fukuyama, Japan; 43 JA Hiroshima General Hospital, Hiroshima, Japan; 44 Kumamoto University Hospital, Kumamoto, Japan; 45 Hachinohe City Hospital, Hachinohe, Japan; 46 Osaka City General Hospital, Osaka, Japan; 47 National Hospital Organization Disaster Medical Center, Tokyo, Japan; 48 University of Toyama, Toyama, Japan; 49 Sapporo Medical University, Sapporo, Japan; 50 Okayama Saiseikai General Hospital, Okayama, Japan; 51 Juntendo University Nerima Hospital, Tokyo, Japan; 52 National Hospital Organization Hokkaido Medical Center, Sapporo, Japan; 53 Akita University Hospital, Akita, Japan; 54 Japanese Red Cross Society Kyoto Daini Hospital, Kyoto, Japan; 55 Maebashi Red Cross Hospital, Maebashi, Japan; 56 Sendai City Hospital, Sendai, Japan; 57 Subaru Health Insurance Society Ota Memorial Hospital, Gunma, Japan; 58 Fukuoka University Hospital, Fukuoka, Japan; 59 Ishikawa Prefectural Central Hospital, Ishikawa, Japan; 60 Shiga University of Medical Science, Shiga, Japan; 61 Nihon University School of Medicine, Tokyo, Japan; 62 Seirei Yokohama General Hospital, Kanagawa, Japan; 63 National Hospital Organization Kumamoto Medical Center, Kumamoto, Japan; 64 Saiseikai Utsunomiya Hospital, Utsunomiya, Japan; 65 National Hospital Organization Higashi-Ohmi General Medical Center, Gifu, Japan; 66 National Hospital Organization Mito Medical Center, ibaraki, Japan; 67 Rinku General Medical Center, Izumisano, Japan

**Keywords:** Bacterial infection, Epidemiology

## Abstract

The clinical implications of bacteremia among septic patients remain unclear, although a vast amount of data have been accumulated on sepsis. We aimed to compare the clinical characteristics and outcomes of severe sepsis patients with and without bacteremia. This secondary analysis of a multicenter, prospective cohort study included 59 intensive care units (ICUs) in Japan between January 2016 and March 2017. The study cohort comprised 1,184 adults (aged ≥ 16 years) who were admitted to an ICU with severe sepsis and diagnosed according to the Sepsis-2 criteria. Of 1,167 patients included in the analysis, 636 (54.5%) had bacteremia. Those with bacteremia had significantly higher rates of septic shock (66.4% vs. 58.9%, p = 0.01) and higher sepsis severity scores, including the Acute Physiology and Chronic Health Evaluation (APACHE) II and the Sequential Organ Failure Assessment (SOFA). No significant difference in in-hospital mortality was seen between patients with and without bacteremia (25.6% vs. 21.0%, p = 0.08). In conclusion, half of severe sepsis patients in ICUs have bacteremia. Although patients with bacteremia had more severe state, between-group differences in patient-centered outcomes, such as in-hospital mortality, have not been fully elucidated.

## Introduction

Sepsis has been defined and recognized as septicemia, which is the invasion and persistence of pathogenic bacteria in the bloodstream^1^. In fact, both sepsis and bacteremia have been a tangled concept until an international consensus definition was developed^2^. Since the concept of sepsis was introduced, a large amount of research has been performed, and sepsis campaign guidelines have been established^3^. However, studies investigating the clinical characteristics and outcomes of bacteremia are rarely reported, even though both bacteremia and sepsis are widespread in critically ill patients^4,5^. Moreover, bacteremia and sepsis are not a nested structure but closely interact with each other^6,7^. The clinical characteristics and implications of bacteremia remain unclear among septic patients^8–10^.

Therefore, we aimed to compare the clinical characteristics and outcomes of severe sepsis patients with and without bacteremia.

## Methods

### Design, setting, and participants

The present study is a secondary analysis of the sepsis cohort in the Focused Outcomes Research in Emergency Care in Acute Respiratory Distress Syndrome, Sepsis and Trauma (FORECAST) study, a multicenter, prospective cohort study of patients with severe sepsis. FORECAST was conducted in 59 intensive care units (ICUs) in Japan between January 2016 and March 2017^11^. Adult patients (aged ≥ 16 years) with severe sepsis, including septic shock, based on Sepsis-2 criteria^12^ and admitted to a participating ICU were included. All patients were selected from the FORECAST database, excluding those with missed or inconsistent blood culture data.

### Data collection

The data collection methods used in the present study were described previously^11^. Briefly, data were extracted from the FORECAST database, including demographics, admission source, various comorbidities, activities of daily living, suspected sites of infection, organ dysfunctions, sepsis-related severity scores, microbiology test results, and information on antibiotic use before arrival. All laboratory data were obtained on arrival at the study hospital. Moreover, we evaluated in-hospital mortality, status after discharge, ventilator-free days (VFDs), ICU-free days (IFDs), and length of hospital stay (LOS).

### Data definition

Severe sepsis was defined according to the Sepsis-2 criteria, that is, patients who met ≥2 systemic inflammatory response syndrome criteria and patients who had at least one organ dysfunction: systolic blood pressure <90 mmHg, mean arterial pressure (MAP) <65 mmHg, or low blood pressure >40 mmHg; serum creatinine >2.0 mg/dL or diuresis (urine output <0.5 mL/kg/h); total bilirubin >2.0 mg/dL; platelet count <100,000 cells/mm^3^; arterial lactate >2 mmoL/L; international normalized ratio >1.5; and arterial hypoxemia (partial pressure of arterial oxygen (PaO2)/fraction of inspired oxygen (FIO2) <200) with pneumonia or PaO2/FIO2 <250 without pneumonia). Blood cultures were performed upon arrival to the ICU and analyzed by local laboratories. Bacteremia was defined as the presence of a positive pathogen (except contamination) in blood culture. Contaminated blood culture results and source of infection were clinically determined by each physician in charge. The source of infection was classified into 11 categories: lung, abdomen, urinary tract, soft tissue, central nervous system, intravenous catheter, osteoarticular, endocardium, wound, implant device, and others. Septic shock was defined according to the Sepsis-2 criteria^12^. VFDs was defined as the number of days within the first 28 days after enrollment during which a patient was able to breathe without a ventilator. VFDs in patients who died during the study period were assigned a score of 0. IFDs were calculated in the same manner as the VFDs.

### Analysis

Continuous variables were presented as median and interquartile range and compared using the Mann–Whitney *U* test because all variables were not normally distributed. Categorical variables were presented as numbers and percentages and compared using either the χ2 or Fisher exact test.

We investigated baseline characteristics such as age, sex, coexisting conditions, sepsis severity, including Acute Physiology and Chronic Health Evaluation (APACHE) II and Sequential Organ Failure Assessment (SOFA) scores, presence of septic shock and acute respiratory distress syndrome, laboratory data, site of infection, and outcomes between severe sepsis patients with and without bacteremia. To identify the association between bacteremia and in-hospital mortality, we performed a univariate analysis and a multivariable logistic regression adjusting age, sex, Charlson comorbidity index, SOFA score, septic shock, and site of infection (lung, abdomen, urinary tract, soft tissue, and others) which were selected based on previous reports^9,10^ and clinical importance. In addition, a subgroup analysis was performed to evaluate the interaction between different pathogenic species and understand the characteristics of bacteremia. We chose six isolated pathogens based on their phylogenetic relationship (*Streptococcus* spp., *Staphylococcus* spp., *Enterococcus* spp., *Escherichia coli*, *Klebsiella* spp., and *Pseudomonas* spp.). Patients in the subgroup analysis were excluded if they had no data on the pathogen, mixed culture results, or inconsistent data about the pathogen because our aim was to describe the characteristics of each pathogen.

For all analyses, a P-value < 0.05 was considered statistically significant. All statistical analyses were performed with EZR (version 1.38; Saitama Medical Center, Jichi Medical University, Saitama, Japan), a graphical user interface for R (version 3.5.0; The R Foundation for Statistical Computing, Vienna, Austria)^13^. More specifically, EZR is a modified version of R commander designed to apply statistical functions frequently used in biostatistics.

### Ethics approval and consent to participate

The study protocol was reviewed and approved by the ethics committees of all institutions that participated in the Japanese Association for Acute Medicine (JAAM) study group. The ethics committees waived a need to obtain informed consent from the study participants, given the retrospective and anonymized nature of this study in the routine care. Institutional Review Board approval (No. 014-0306) was granted by Hokkaido University, the lead institution for FORECAST. All methods were carried out in accordance with relevant guidelines and regulations. The ethics committees at all institutions approved that this subgroup analysis also waived the need for informed consent.

## Results

### Clinical characteristics

The present study included 1,167 patients with severe sepsis among 1,184 patients enrolled in the FORECAST database. The median age was 73 years [interquartile range (IQR): 64–81], and 458 patients were female (39.2%). Among the included patients, 636 (54.5%) had bacteremia. Bacteremia was less prevalent in males compared with females [359 (56.4%) vs. 350 (65.9%), p < 0.01; Table 1]. Patients with bacteremia had significantly higher rates of septic shock compared with those without bacteremia [422 (66.4%) vs. 313 (58.9%), p = 0.01]. Charlson comorbidity index was not different in patients with bacteremia versus those without bacteremia [1 (0–2) vs. 1 (0–2), p = 0.55]. No significant difference was noted for most comorbidities among the groups. Chronic obstructive pulmonary disease was less widespread in those with versus without bacteremia [33 (5.2%) vs. 49 (9.2%), p = 0.01]. Sepsis severity scores, such as the APACHE II and SOFA, were higher in patients with versus without bacteremia [23 (18–30) vs. 22 (16–29), p = 0.02 and 9 (6–12) vs. 8 (5–11), p < 0.01, respectively]. Patients with bacteremia had a higher rate of most types of organ dysfunction when compared with those without bacteremia. Those with bacteremia were less likely to have acute lung injury compared with those without bacteremia [196 (30.8%) vs. 240 (45.2%), p < 0.01]. Laboratory data revealed lower white blood cell and platelet counts in patients with compared with without bacteremia [10,600 (4,800–17,300)/µL vs. 12,400 (6,600–18,400)/µL, p < 0.01, and 12.4 (7.5–18.6) × 10^4^/µL vs. 17.5 (11.1–24.5) × 10^4^/µL, p < 0.01, respectively]. In addition, patients with bacteremia had significantly higher median lactate level (3.3 vs. 2.6 mmol/L), C-reactive protein (CRP) level (16.5 vs. 15.3 mg/dL), and serum procalcitonin (PCT) concentration (21.3 vs. 5.2 ng/mL; p < 0.01, p = 0.02, and p < 0.01, respectively). Regarding to site of infection, patients with bacteremia were less likely to have lung infection and more likely to have urinary tract infection compared with those without bacteremia [133 (20.9%) vs. 299 (43.1%), p < 0.01 and 160 (25.2%) vs. 56 (10.5%), p < 0.01, respectively].

**Table 1:** Characteristics comparison between patients with and without bacteremia (n = 1,167).

		BC positive (n = 636)	BC negative (n = 531)	P-value
Age		73 (65–82)	72 (63–81)	0.11
Sex (male)		359 (56.4)	350 (65.9)	<0.01
Coexisting conditions	Myocardial infarction	27 (4.2)	30 (5.6)	0.33
	Congestive heart failure	59 (9.3)	68 (12.8)	0.07
	Peripheral vascular disease	14 (2.2)	15 (2.8)	0.62
	Cerebrovascular disease	72 (11.3)	66 (12.4)	0.59
	Dementia	53 (8.3)	41 (7.7)	0.78
	COPD	33 (5.2)	49 (9.2)	0.01
	Connective tissue disease	43 (6.8)	39 (7.3)	0.78
	Peptic ulcer disease	17 (2.7)	14 (2.6)	>0.99
	Diabetes mellitus without organ damage	107 (16.8)	88 (16.6)	0.97
	Diabetes mellitus with organ damage	49 (7.7)	26 (4.9)	0.07
	Chronic kidney disease	49 (7.7)	35 (6.6)	0.54
	Hemiplegia	20 (3.1)	23 (4.3)	0.36
	Malignancy (solid)	81 (12.7)	60 (11.3)	0.51
	Malignancy (blood)	8 (1.3)	14 (2.6)	0.13
	Metastatic tumor	13 (2.0)	13 (2.4)	0.79
	Mild liver disease	31 (4.9)	15 (2.8)	0.10
	Moderate to severe liver disease	16 (2.5)	10 (1.9)	0.60
	AIDS	1 (0.2)	0	>0.99
CCI		1 (0–2)	1 (0–2)	0.55
ADL (dependent)		163 (25.6)	120 (22.7)	0.27
Septic shock		422 (66.4)	313 (58.9)	0.01
APACHE II score		23 (18–30)	22 (16–29)	0.02
SOFA score		9 (6–12)	8 (5–11)	<0.01
Organ dysfunction on arrival	Hypotension	373 (58.6)	275 (51.8)	0.02
	Hyperbilirubinemia (>2.0 mg/dL)	132 (20.8)	70 (13.2)	<0.01
	Acute kidney injury (Cre>2 mg/dL)	259 (40.7)	191 (36.0)	0.11
	Acute lung injury	196 (30.8)	240 (45.2)	<0.01
	Hyperlactatemia (>2 mmol/L)	458 (72.0)	327 (61.6)	<0.01
	Thrombocytopenia (<100,000/μl)	241 (37.9)	98 (18.5)	<0.01
	Coagulopathy (INR >1.5)	131 (20.6)	90 (16.9)	0.13
White blood cells (/µL)		10600 (4800–17300)	12400 (6600–18400)	<0.01
Hematocrit (%)		33.9 (28.9–39.1)	34.2 (28.9–39.7)	0.35
Platelet (/µL)		12.4 (7.5–18.6)	17.5 (11.1–24.5)	<0.01
Total protein (g/dL)		5.7 (5.0–6.4)	5.8 (5.0–6.5)	0.21
Serum albumin (g/dL)		2.6 (2.1–3.1)	2.6 (2.2–3.2)	0.29
Creatinine (mg/dL)		1.6 (1.0–2.5)	1.4 (0.9–2.6)	0.05
Total bilirubin (mg/dL)		1.0 (0.6–1.8)	0.8 (0.5–1.3)	<0.01
Lactate (mmol/L)		3.3 (2.1–5.6)	2.6 (1.6–4.7)	<0.01
C-reactive protein (mg/dL)		16.5 (8.8–25.5)	15.3 (6.3–24.3)	0.02
Procalcitonin (ng/mL)		21.3 (3.5–75.6)	5.2 (1.1–34.4)	<0.01
Source of infection	Lung	133 (20.9)	229 (43.1)	<0.01
	Abdomen	154 (24.2)	151 (28.4)	0.11
	Urinary tract	160 (25.2)	56 (10.5)	<0.01
	Soft tissue	67 (10.5)	50 (9.4)	0.60
	Central nervous system	16 (2.5)	6 (1.1)	0.09
	Intravenous catheter	20 (3.1)	2 (0.4)	<0.01
	Osteoarticular	17 (2.7)	4 (0.8)	0.01
	Endocardium	13 (2.0)	3 (0.6)	0.04
	Wound	8 (1.3)	4 (0.8)	0.56
	Implant device	7 (1.1)	1 (0.2)	0.08
	Other	41 (6.4)	25 (4.7)	0.25

Reported counts (proportions) for categorical variables and median (interquartile range) for continuous variables.

Continuous variables were compared using the Mann–Whitney U test. Categorical variables were compared using the Fisher’s exact test or chi square test, where appropriately.

Missing data (BC positive/BC negative); ADL (0/2), Mechanical ventilation (23/10), APATCH II score (97/62), SOFA score (113/67), White blood cells (2/3), Hematocrit (2/3), Platelet (2/3), Total protein (29/20), Serum albumin (17/11), Creatinine (3/4), Total bilirubin (8/8), Lactate (20/15), C-reactive protein (9/8), Procalcitonin (282/257).

COPD: Chronic obstructive pulmonary disease, AIDS: Acquired immunodeficiency syndrome, CCI: Charlson comorbidity index, ADL: Activities of daily living, APACHE: Acute physiology and chronic health evaluation, SOFA: Sequential organ failure assessment, BC: Blood culture.

### Patient-centered outcomes

In-hospital mortality was not significantly different between those with and without bacteremia [158/618 (25.6%) vs. 108/515 (21.0%), p = 0.08; Table 2]. However, among patients with shock, in-hospital mortality was significantly higher in those with versus without bacteremia [128/410 (31.2%) vs. 70/300 (23.3%), p = 0.03]. Among the groups, no significant difference was observed in VFDs, IFDs, or LOS. Bacteremia was not associated to in-hospital mortality in an unadjusted analysis (odds ratio 1.29 [95% confidential interval, 0.98–1.71], p = 0.07) or in an adjusted analysis (odds ratio 1.16 [95% confidential interval 0.82–1.63], p = 0.41; Table 3).

**Table 2:** Outcomes and disposition among patients with and without bacteremia.

		BC positive (n = 636)	BC negative (n = 531)	P-value
In-hospital mortality	All	158 (25.6)	108 (21.0)	0.08
	With shock	128 (31.2)	70 (23.3)	0.03
	Without shock	30 (14.4)	38 (17.7)	0.44
Survivor dispositions	Home	181 (39.3)	139 (34.2)	0.13
	Transfer	279 (60.7)	268 (65.8)	
ICU-free days		20 (11–24)	19 (10–24)	0.10
Ventilator-free days		21 (0–28)	21 (0–27)	0.55
Length of hospital stay		23 (12–44)	24 (13–50)	0.10

Reported counts (proportions) for categorical and median (interquartile range) for continuous variables.

Continuous variables were compared using the Mann–Whitney U test. Categorical variables were compared using the Fisher’s exact test or chi square test, where appropriately.

Missing data (BC positive/BC negative); In-hospital mortality (18/16), In-hospital mortality with shock (12/13), In-hospital mortality without shock (6/3), Survivor dispositions (18/16), ICU-free days (153/106), Ventilator-free days (26/19), Length of hospital stay (18/16).

ICU: Intensive care unit, BC: Blood culture.

**Table 3:** The association between patients with and without bacteremia and in-hospital mortality.

Covariables		Odds ratio	95% confidence interval	P-value
Bacteremia	Unadjusted	1.29	0.98–1.71	0.07
	Adjusted	1.16	0.82–1.63	0.41

Adjusted: age, sex, Charlson comorbidity index, SOFA score, septic shock, and site of infection (lung, abdomen, urinary tract, soft tissue, and others).

SOFA: SOFA: Sequential organ failure assessment.

### Characteristics and outcomes according to pathogenic species

Among 636 patients with bacteremia, 403 patients were identified as having the most common six pathogen species. Then, we described the 403 patients in the details as a subgroup analysis (Table 4). Among those, the most prevalent pathogen was *E. coli* [157 (39.0%)], followed by *Streptococcus* [92 (22.8%)] and *Staphylococcus* [89 (22.1%)]. *E. coli* was associated with the lowest in-hospital mortality [22/154 (14.3%)]. Sepsis severity scores, such as the APACHE II and SOFA scores, were heterogeneous, and organ dysfunction depended on the type of pathogen. Laboratory data revealed that inflammatory biomarkers such as CRP and PCT varied among pathogenic species. The highest CRP concentration was observed among those with *Streptococcus* [25.5 (14.8–33.0) mg/dL]. The highest PCT level was identified in *Klebsiella* [68.4 (21.3–132.1) ng/mL], followed by *E. coli* [28.0 (4.2–95.5) ng/mL], and the lowest PCT level was found in *Staphylococcus* [7.1 (1.6–22.8) ng/mL].

**Table 4:** Subgroup analysis of relationship between each pathogen and variables among patients with bacteremia (n = 403).

		GPC			GNR		
		*Streptococcus* (n = 92)	*Enterococcus* (n = 10)	*Staphylococcus* (n = 89)	*Escherichia coli* (n = 157)	*Klebsiella* (n = 47)	*Pseudomonas* (n = 8)
Septic shock		47 (51.1)	8 (80.0)	57 (64.0)	100 (63.7)	38 (80.9)	6 (75.0)
APACHE II score		22 (17–31)	24 (21–26)	26 (18–33)	22 (18–28)	25 (20–32)	30 (26–35)
SOFA score		9 (5–12)	9 (7–11)	9 (7–12)	9 (6–12)	12 (10–13)	11 (8–12)
Organ dysfunction on arrival	Hypotension	47 (51.1)	4 (40.0)	47 (52.8)	90 (57.3)	33 (70.2)	5 (62.5)
	Hyperbilirubinemia (>2.0 mg/dL)	19 (20.7)	2 (20.0)	20 (22.5)	24 (15.3)	17 (36.2)	0
	Acute kidney injury (Cre>2 mg/dL)	32 (34.8)	1 (10.0)	35 (39.3)	76 (48.4)	21 (44.7)	1 (12.5)
	Acute lung injury	30 (32.6)	4 (40.0)	26 (29.2)	37 (23.6)	21 (44.7)	4 (50.0)
	Hyperlactatemia (>2 mmol/L)	68 (73.9)	6 (60.0)	62 (69.7)	116 (73.9)	39 (83.0)	7 (87.5)
	Thrombocytopenia (<100,000/μl)	38 (41.3)	2 (20.0)	38 (42.7)	60 (38.2)	17 (36.2)	2 (25.0)
	Coagulopathy (INR >1.5)	20 (21.7)	2 (20.0)	16 (18.0)	32 (20.4)	14 (29.8)	2 (25.0)
White blood cells (/µL)		8100 (3300–13000)	12600 (9100–18200)	12100 (8000–17300)	11200 (5000–17300)	10700 (4900–17600)	3200 (900–16100)
Hematocrit (%)		36.6 (30.1–41.4)	34.4 (30.5–39.6)	34.6 (30.2–40.3)	33.1 (28.1–38.8)	33.4 (29.6–37.4)	31.6 (27.4–35.8)
Platelet (/µL)		11.4 (6.4–19.2)	23.3 (15.7–30.7)	12.2 (7.5–20.1)	12.4 (7.8–16.8)	11.6 (6.9–14.2)	16.0 (2.7–23.3)
Total protein (g/dL)		5.8 (5.3–6.6)	5.7 (4.9–6.5)	6.0 (5.2–6.8)	5.8 (5.3–6.4)	5.4 (4.7–6.0)	4.8 (4.1–5.3)
Serum albumin (g/dL)		2.6 (2.2–3.2)	2.4 (2.0–2.7)	2.6 (2.0–3.2)	2.8 (2.3–3.3)	2.5 (2.1–2.9)	2.3 (2.1–2.5)
Creatinine (mg/dL)		1.6 (1.0–2.4)	1.4 (0.9–1.5)	1.7 (1.0–2.9)	1.7 (1.0–2.5)	1.7 (1.1–2.9)	1.2 (0.5–1.5)
Total bilirubin (mg/dL)		1.1 (0.8–1.6)	0.9 (0.4–1.9)	1.0 (0.6–1.9)	1.0 (0.7–1.6)	1.5 (0.7–4.1)	0.8 (0.7–1.2)
Lactate (mmol/L)		3.2 (2.1–6.2)	2.5 (1.4–2.8)	2.9 (2.1–5.1)	3.6 (2.3–5.5)	4.5 (2.7–6.6)	3.5 (2.5–5.1)
C-reactive protein (mg/dL)		25.5 (14.8–33.0)	8.7 (5.8–16.7)	17.1 (7.5–28.7)	15.5 (8.3–22.3)	14.9 (9.3–25.4)	11.4 (7.1–16.1)
Procalcitonin (ng/mL)		24.3 (4.9–71.3)	16.2 (4.2–30.7)	7.1 (1.6–22.8)	28.0 (4.2–95.5)	68.4 (21.3–132.1)	17.3 (2.7–31.0)
In-hospital mortality		22 (24.4)	3 (30.0)	30 (34.5)	22 (14.3)	11 (24.4)	3 (37.5)

Reported counts (proportions) for categorical and median (interquartile range) for continuous variables.

Missing data; Total protein = 15, Serum albumin = 7, Total bilirubin = 2, Lactate = 14, C-reactive protein = 3, Procalcitonin = 184, In-hospital mortality = 9, APACHE II score = 67, SOFA score = 76.

GPC: Gram-positive cocci, GNR: Gram-negative rods, APACHE: Acute physiology and chronic health evaluation, SOFA: Sequential organ failure assessment.

## Discussion

### Brief summary

We evaluated the clinical characteristics and outcomes of severe sepsis patients with and without bacteremia. Bacteremia was present in half of the patients with severe sepsis. Outcomes, including in-hospital mortality, VFDs, IFDs, and LOS, were not significantly different among severe sepsis patients with and without bacteremia, although the severity scores were higher in those with bacteremia.

### Characteristics and outcomes of severe sepsis patients with and without bacteremia

Several studies have reported on the epidemiology of bacteremia and sepsis, although the quality of such studies is varied^5,9,10,14–17^. In the present study, higher rates of septic shock were seen in patients with versus without bacteremia. It is well known that the amount and type of cytokines differ in their severity. For example, cytotoxic cytokines, such as tumor necrosis factor-α, are released more frequently in cases of bacteremia^7^. The variety in the production of cytokines that stimulate inflammation may explain the prevalence of septic shock in the bacteremia group. Moreover, detection of bacteremia depends on the amount of bacteria. Bacteremia (amount of bacteria) may have correlated with prognosis or exacerbation of sepsis because there were more septic patients with shock in patients with bacteremia than in those without bacteremia^8^.

However, in-hospital mortality was not significantly different between patients with and without bacteremia in our study. Previous studies have reported various mortality rates in patients with bacteremia^6–8,10,15^. The diversity of the study population, setting, and study design may have contributed to the different impacts of bacteremia, which may have been influenced by the proportion of patients with each pathogenic species in each study^5,14^, or evidence of pathogen may have aided in selecting the appropriate antibiotic^16^. Patients with sepsis and bacteremia may have received broad-spectrum antibiotics earlier when compared with patients without bacteremia because patients with bacteremia were in more severe state^9,16^. Alternatively, as reported in our study, only the most severe cases of bacteremia, such as those in shock, had a higher mortality compared with those without bacteremia. Further studies are needed to assess the effect of bacteremia on patient outcomes.

Patients with bacteremia had higher concentrations of inflammatory markers, such as PCT, than did those without bacteremia. In the subgroup analysis, in-hospital mortality and inflammatory markers such as PCT varied according to pathogenic species. No clear mechanism exists for these differences in pathogenicity; however, the differences may be associated with the differences in intracellular pathways and cytokines^18^. A previous study reported that patients with gram-negative bacteremia had higher PCT levels compared with those with gram-positive bacteremia^6^. This may be due to a cell wall component, wherein lipopolysaccharides are found in gram-negative bacteria and lipoteichoic acid is found in gram-positive bacteria^19^. It may be difficult to use PCT alone to predict the pathogenic species in the development of sepsis, although it is useful for differentiating gram-negative from gram-positive bacteremia^6^. In addition, severity of sepsis and poor renal function influenced elevated PCT levels^20,21^. Further studies are needed to investigate the impact of PCT level in choosing appropriate treatments.

### Limitations

Our study has some limitations. First, only ICU patients in Japan were enrolled. Our population was relatively old with severe disease, which may account for a higher proportion of bacteremia in Japan when compared with other countries^5,6^. Second, there were large missing data regarding PCT, which might be affected the results. Third, we did not define the blood culturing methods such as the volume of blood per culture or the methods used to detect bacteremia in each institution. There might have been misclassification with respect to blood culture results because we did not standardize the blood culture methods. This misclassification might have caused underestimation of the association between the presence of bacteremia and clinical outcomes. However, there may be less chance of misclassification because our practice in Japan was relatively homogenous and the compliance rate of obtaining blood cultures was very high^11^. Fourth, we did not follow up the patients after they were discharged from the hospital. Because our outcome was in-hospital mortality, we can only assume that some patients died after being discharged from the hospital. Fifth, the contamination and site of infection were determined based on each physician’s judgement. However, there would not be much difference because clinical practices in Japan back then were relatively uniform^11^. Sixth, we categorized pathogens broad major groups such as *Staphylococcus* spp. regardless of their species or virulence because of small number of each pathogen. Finally, we did not have information about antimicrobial susceptibility and antimicrobial resistance data. However, our previous research^11^ showed that the majority of antibiotics we used were broad-spectrum antibiotics such as carbapenems and high compliance with 3-h bundles. In addition, all participating institutions were national-certified emergency centers and intensive care units. Therefore, we believe that most patients received appropriate treatments.

## Conclusions

Half of the patients with severe sepsis in the present study had positive blood culture results. According to the sepsis severity scores, septic patients with bacteremia were more severe than those without bacteremia. Moreover, those with bacteremia had high inflammatory markers such as PCT. However, between-group differences in patient-centered outcomes, such as in-hospital mortality, remain unclear.

## Data Availability

The datasets during and/or analyzed during the current study available from the corresponding author on reasonable request.
